# Associations of person-related, environment-related and communication-related factors on medication errors in public and private hospitals: a retrospective clinical audit

**DOI:** 10.1186/s12913-021-07033-8

**Published:** 2021-09-28

**Authors:** Elizabeth Manias, Maryann Street, Grainne Lowe, Jac Kee Low, Kathleen Gray, Mari Botti

**Affiliations:** 1 School of Nursing and Midwifery, Centre for Quality and Patient Safety Research, Institute for Health Transformation, Deakin University, 221 Burwood Highway, Burwood, Victoria 3125 Australia; 2 Centre for Digital Transformation of Health, The University of Melbourne, Grattan Street, Parkville, Victoria 3010 Australia

**Keywords:** Family, Health communication, Medication errors, Medication therapy management, Patient safety, Patient participation

## Abstract

**Background:**

Efforts to ensure safe and optimal medication management are crucial in reducing the prevalence of medication errors. The aim of this study was to determine the associations of person-related, environment-related and communication-related factors on the severity of medication errors occurring in two health services.

**Methods:**

A retrospective clinical audit of medication errors was undertaken over an 18-month period at two Australian health services comprising 16 hospitals. Descriptive statistical analysis, and univariate and multivariable regression analysis were undertaken.

**Results:**

There were 11,540 medication errors reported to the online facility of both health services. Medication errors caused by doctors (Odds Ratio (OR) 0.690, 95% CI 0.618–0.771), or by pharmacists (OR 0.327, 95% CI 0.267–0.401), or by patients or families (OR 0.641, 95% CI 0.472–0.870) compared to those caused by nurses or midwives were significantly associated with reduced odds of possibly or probably harmful medication errors. The presence of double-checking of medication orders compared to single-checking (OR 0.905, 95% CI 0.826–0.991) was significantly associated with reduced odds of possibly or probably harmful medication errors. The presence of electronic systems for prescribing (OR 0.580, 95% CI 0.480–0.705) and dispensing (OR 0.350, 95% CI 0.199–0.618) were significantly associated with reduced odds of possibly or probably harmful medication errors compared to the absence of these systems. Conversely, insufficient counselling of patients (OR 3.511, 95% CI 2.512–4.908), movement across transitions of care (OR 1.461, 95% CI 1.190–1.793), presence of interruptions (OR 1.432, 95% CI 1.012–2.027), presence of covering personnel (OR 1.490, 95% 1.113–1.995), misread or unread orders (OR 2.411, 95% CI 2.162–2.690), informal bedside conversations (OR 1.221, 95% CI 1.085–1.373), and problems with clinical handovers (OR 1.559, 95% CI 1.136–2.139) were associated with increased odds of medication errors causing possible or probable harm. Patients or families were involved in the detection of 1100 (9.5%) medication errors.

**Conclusions:**

Patients and families need to be engaged in discussions about medications, and health professionals need to provide teachable opportunities during bedside conversations, admission and discharge consultations, and medication administration activities. Patient counselling needs to be more targeted in effort to reduce medication errors associated with possible or probable harm.

## Background

Medication management is a fundamental activity of health care encompassing prescribing, dispensing, preparing, administering and monitoring medications. Safe and optimal delivery of medications requires health professionals, patients and family members to have a clear understanding of the potential harm that medications can cause, and of how safe practices can assist in reducing the prevalence of medication errors. Medication management involves working across diverse interfaces, comprising interactions between health professionals, within and across clinical settings, and traversing time and location. All these medication management activities require clear and accurate documentation. Documentation processes include ordering of medications on prescription charts, identifying medications have been administered by providing the nurses’ signature, and writing patients’ therapeutic and adverse responses to medications in the medical records. The need to complete documentation repeatedly, by diverse individuals in different situations, puts patients at risk of medication errors [1]. In examining the nature of medication errors, extensive research has focused on identifying the prevalence, types and severity of medication errors [2–6]. Other important considerations comprise examining how person-related, environment-related and communication-related factors are associated with medication errors.

Person-related factors relate to the health professionals managing medications, and the patients in their care. When medication errors occur, a variety of health profession disciplines may be responsible for causing these errors, including nurses, doctors and pharmacists. Various individuals are also involved in reporting these medication errors, and they may not necessarily be the same individuals who are responsible for causing the medication errors. Also crucial is the role of patients and family members in communicating with health professionals about preventing a medication error from occurring and identifying a medication error once it has occurred [7]. Individuals confronted by stress and intimidating behaviour can also affect medication errors [8]. While past studies have considered who was responsible for, or contributed to, the occurrence of the medication error, there has been little emphasis on patient and family involvement [9–11].

Environment-related factors include patients’ movements across settings, changes of shift situations and effects of interruptions. The environment is also influenced by various policies and protocols to be understood and followed by health professionals [12]. Past work has shown that an increase in the number of ward transfers was associated with higher odds of prescribing errors [13], or the increased prevalence of communication failure relating to the management of medications [14].

Communication-related factors comprise the various structures situated in clinical settings by which health professionals convey information. These structures may be formal, such as clinical handover, telephone orders for medications and ward rounds. Formal structures of communication are predetermined and planned events, scheduled to occur at set times and require a particular process to be followed. Communication may also be informal such as ad hoc corridor interactions, and informal bedside conversations. Informal structures of communication are not pre-planned, may occur at any time, and do not require a particular process to be followed. Communication-related factors also include the various channels of communication involving medication orders, such as electronic and paper based systems. Safe and effective medication management can be facilitated through communicating with electronic systems [15, 16]. In an overview of reviews, electronic systems were shown to reduce medication errors by enhancing communication between health professionals, and enabling ready access to guidelines on disease and treatment monitoring [17]. This overview also showed that electronic systems have the potential to increase accuracy and completeness of clinical information and to reduce health professional documentation time. In a retrospective audit of medication incident reports, investigators examined how communication problems contributed to 500 reported medication errors [18]. Common communication problems related to digital communication, such as missing medication information in patients’ computerised records (68.2%; *n* = 341), and lack of interaction within a health care team, such as insufficient or inaccurate information conveyed at shift changes (39.6%; *n* = 198). Other communication problems were health professionals’ misunderstandings about clinical activities of temporary and permanent health professionals (25.6%; *n* = 128).

The aim of this study was to determine the associations of person-related, environment-related and communication-related factors on the severity of medication errors occurring in two health services. This study fills a gap in the literature by providing enhanced understandings about the characteristics of person-related, environment-related and communication-related factors in relation to reported medication errors. Also important is to consider how these factors are associated with patient harm arising from medication errors. The knowledge gained will inform the development of future targeted and tailored guidelines for improved safety and quality of care, with the potential to reduce medication errors.

## Methods

### Study design

A retrospective clinical audit was undertaken of reported medication errors occurring at two Australian health services in the state of Victoria. Health professionals submitted the details of these medication errors to an on-line voluntary incident reporting system. While the reporting system for medication errors was a voluntary process, this activity was a crucial part of patient safety governance in the health services.

The key health professionals involved in medication management in both health services were registered nurses, endorsed enrolled nurses, registered midwives, registered pharmacists and registered doctors. Registered nurses completed a three-year university degree course and were responsible for preparing, administering and monitoring medications. Endorsed enrolled nurses completed an 18-month diploma course and work under the direction of registered nurses. They were also permitted to administer medications under supervision. Midwives were registered health professionals who completed either a combined four-year nursing and midwifery university course, a standalone three-year midwifery university course, or a postgraduate midwifery university course. Midwives were responsible for preparing, administering and monitoring medications in situations relating to ante-natal and post-natal care. Registered pharmacists completed a four-year university course followed by a one-year internship. They checked patients’ medication charts upon patient admission and reconciled all medications as patients moved between settings. They conducted patient and family medication counselling, provided expert advice to other health professionals about medications prescribed and administered, and supplied ordered medications to clinical settings. Registered doctors completed at least a five- to six-year university degree and a one-year internship. They were responsible for prescribing medications and monitoring for therapeutic and adverse effects. In view of the medication activities of these health professional disciplines, they were also the major ones contributing to medication errors.

Ethics approval was obtained from both health services and by the Deakin University Human Research Ethics Committee (DUHREC) (approval numbers: LR19/2017, EH2017–185, and DU2018–063) to enable conduct of the clinical audit. Informed consent was waived by the ethics committees for conduct of the research in view of the retrospective design of the project. All methods were carried out in reference to the National Statement on Ethical Conduct in Human Research [19].

One health service was a private, non-profit organisation comprising nine hospitals, while the other was a public organisation involving seven hospitals, providing care for patients. Thus, 16 hospitals were included in the audit. These hospitals were situated in diverse metropolitan and regional areas and comprised a mixture of tertiary care, community care, acute care, and geriatric rehabilitation facilities. After obtaining ethics approval, details about the medication errors were retrieved by liaising with a safety and quality officer at each health service site. Each safety and quality officer removed any identifiable information from the records, such as patients’ and health professionals’ names before providing the database to the research team through a secure drive.

Inclusion criteria were all medication errors reported within each health service from the 1st of October, 2015 to the 31st of March, 2017. To accompany provision of information using check-box responses, health professionals also documented descriptions about medication errors in the form of free-text. There were no exclusion criteria for this study.

### Data collection

The on-line voluntary system used by health services provided a standardised framework for collecting and classifying medication errors. Within each health service, health professionals were actively encouraged by hospital managers to report medication errors on the on-line system. The safety and quality officer at each health service site filtered the required eligibility criteria within the online database to obtain the required sample. For this study, a medication error was defined as any preventable event that may cause or lead to inappropriate medication use or patient harm. Medication errors could occur at any stage of the medication management process; supplying, prescribing, preparing, administering and monitoring. Information for reported medication errors could only be submitted if health professionals completed all fields within the online database. Thus, there was no missing information in the data obtained from reported medication errors.

Medication error reports for the designated 18-month period were placed into an Excel spreadsheet. Data comprised the date and time of the error, the patient’s gender, age, medication involved in the error, type of error involved, severity of medication error classification according to the medication safety committee of the hospital, any follow up that occurred after detection and recording of the medication error, and the description of the medication error. The description provided for each medication error was read carefully, regardless of the length of the text and quality of description provided. In reading through the descriptions of medications, there were no entries that were identified as incomprehensible. The research team had access to patients’ medical records if any information required validation. The data were then transported in an IBM SPSS Statistics for Windows, Version 26 (IBM Corp., Armonk, N.Y., USA) database for data cleaning and categorisation according to the categories designated on the National Coordinating Council for Medication Error Reporting and Prevention tool [20]. This tool provides a standardised, systematic and comprehensive approach to recording, tracking and evaluating medication errors. The tool is divided into seven components, comprising the medication error event, patient outcome, description of the medication involved, personnel involved in causing and reporting the error, type of medication error, causes, and contributing factors. The tool facilitated the systematic classification of person-related, environment-related and communication-related factors.

### Data analysis

Descriptive analyses were undertaken, including frequency counts and percentages for all variables of interest, such as types of medication involved in the medication errors, causes and contributing factors. Means and standard deviations were calculated for continuous variables that were normally distributed. Medians and inter-quartile ranges were calculated for continuous variables that were not normally disturbed.

Univariate associations were examined between person-related, environment-related and communication-related factors and severity of medication errors using cross tabulations and chi square tests. The outcome variable was the severity of the medication error in causing harm, according to nine possible outcomes, in accordance with the National Coordinating Council for Medication Error Reporting and Prevention tool [20]. These nine outcomes for severity of harm related to medication errors that were not associated with patient harm and those that were associated with patient harm. The three outcomes that were not associated with patient harm comprised: (1) the circumstances had the capacity to cause a medication error; (2) the medication error occurred but it did not reach the patient; and (3) the medication error occurred and reached the patient, but it did not cause harm. The six outcomes associated with patient harm were the following: (4) the medication error reached the patient, and it required monitoring or intervention to confirm the patient experienced no harm; (5) the medication error may have resulted in temporary harm and required an intervention; (6) the medication error may have resulted in temporary harm and required prolonged hospitalisation; (7) the medication error may have resulted in permanent patient harm; (8) the medication error may have required an intervention necessary to sustain life; and (9) the medication error may have contributed to or resulted in the patient’s death.

For logistic regression analysis, severity of harm was categorised into two possible outcomes and was based on the previously defined nine outcomes for severity of harm. These two outcomes were calculated by collating the frequency counts for scores of 1–3 relating to no patient harm from medication errors and the frequency counts for scores of 4–9 relating to possible or probable patient harm. Person-related factors included the individual who was documented as being responsible for the error (nurse/midwife/doctor/pharmacist/patient/family member), patients’ age (65 years or older/younger than 65 years), involvement of the patient and family in identifying the medication error (yes/no) and the presence of counselling for the patient (yes/no). Environment-related factors included patient movement across transitions of care (yes/no), the presence of interruptions contributing to the medication error (yes/no), and the checking process used by nurses or midwives to administer the medications (single-checking/double-checking). Communication-related factors included having a misread or unread medication order (yes/no), informal bedside conversation (yes/no), clinical handover problem (yes/no), prescribing processes using electronic systems (yes/no), electronic dispensing processes (yes/no), and electronic systems to administer the medication (yes/no). Binary multivariable logistic regression modelling was then undertaken using all explanatory variable factors. The level of significance used for logistic regression modelling was α = 0.05.

## Results

### Characteristics of medication errors

Over the 18-month study period, there were 11,540 medication errors reported to the online facilities of both health services (Table 1). There were 3683 medication errors (31.9%) with a severity of patient harm score of 1–3, indicating that no harm occurred, while there were 7857 medication errors (68.1%) with a severity of patient harm score of 4–9, indicating that possible or probable harm occurred. In relation to patients’ age, older patients were significantly more likely to experience harm from medication errors. For those aged 65 years and above, there were 1861 medication errors where no harm occurred (28.1%) while there were 4759 medication errors related to possible or probable harm (71.9%). Conversely, for people aged younger than 65 years, while there were 1822 medication errors where no harm occurred (37.0%), there were 3098 medication errors related to possible or probable harm (63.0%), χ^2^ = 103.37, *p* < 0.001. The top three clinical settings for medication errors were general medicine (*n* = 3233, 28.0%), psychiatry (*n* = 1255, 10.9%) and rehabilitation (*n* = 1175, 10.2) (Table 1).

**Table 1:** Demographic information about participating health services and clinical settings where medication errors occurred (*N* = 11,540)

**Demographic information of public health service (seven hospitals)**		
Total number of beds		1500
Number of beds at each hospital		25–650
Number of admissions and presentations annually		500,000
Percentage of patients from non-English backgrounds		22
Key services include: emergency care, general medicine general surgery,, geriatric care, gynaecology, intensive care, medical oncology, mental health, obstetrics, orthopaedic surgery, outpatient care, rehabilitation, paediatrics and rehabilitation		
**Demographic information of private health service (nine hospitals)**		
Total number of bed		1550
Number of beds at each hospital		25–700
Number of admissions and presentations annually		240,000
Percentage of patients from non-English backgrounds		15
Key services include: emergency care, general medicine general surgery, gynaecology, intensive care, medical oncology, mental health, orthopaedic surgery, outpatient care, plastic and reconstructive health, rehabilitation, renal dialysis and rehabilitation and urology		
**Clinical setting**	**n**	**%**
General medical unit	3233	28.0
Psychiatric unit	1255	10.9
Rehabilitation unit	1175	10.2
Oncology/Haematology unit	858	7.4
Pharmacy department	721	6.2
Emergency department	628	5.4
General surgical unit	607	5.3
Orthopaedics unit	442	3.8
Obstetrics/gynaecology unit	419	3.6
Cardiac/cardiothoracic unit	357	3.1
Renal/nephrology unit	266	2.3
Gastrointestinal unit	203	1.8
Vascular unit	200	1.7
Respiratory unit	171	1.5
Neurology unit	169	1.5
Plastics unit	161	1.4
Intensive care unit	158	1.4
Urology unit	134	1.2
Neurosurgical unit	121	1.0
Paediatric unit	66	0.6
Perioperative department	49	0.4
Outpatients department	48	0.4
Hospital in the Home	47	0.4
Radiology unit	34	0.3
Immunology and rheumatology unit	10	0.09
Short stay unit	6	0.05
Ophthalmology unit	2	0.02

The median age of patients affected by medication errors was 70.0 years (IQR = 52.0, 82.0). Of the reported medication errors, 53.7% occurred in female patients. Most medication errors occurred during weekdays (*n* = 9091, 78.8%) compared to weekends, indicating an alignment with the proportion of weekdays to weekend days. The majority of medication errors occurred between 0700 and 1530 h (*n* = 7041, 61.0%), but medication errors also occurred from 1531 to 2130 (*n* = 3234, 28.0%) and during the hours of 2131 to 0659 (*n* = 1265, 11.0%).

Single-checking of medications was documented in 8271 (71.7%) medication errors while double-checking was documented in 3269 (28.3%) medication errors. The individuals reporting the medication errors were largely nurses or midwives (*n* = 8618, 74.7%), compared with pharmacists (*n* = 2519, 21.8%) and doctors (*n* = 205, 1.8%). In regards to individuals responsible for medication errors, nurses or midwives were most commonly responsible (*n* = 8303, 71.9%), followed by doctors (2525, 21.9%) and pharmacists (*n* = 443, 3.8%). Patients and families were also responsible for 269 (2.4%) medication errors. Health professionals who were not nurses or midwives reported 23.6% of medication errors, and were responsible for 25.7% of medication errors. Furthermore, while doctors were responsible for about 20% (2519/11,540) of medication errors, they reported only about 2% (205/11,540) of these errors.

### Severity of patient harm relating to medication errors

Table 2 shows the breakdown of severity of patient harm relating to medication errors. The most common patient outcome was a medication error occurred that reached the patient, and it required monitoring and intervention to confirm if no harm had actually occurred (*n* = 7095, 61.5%). Other common patient outcomes were the medication error occurred but it did not reach the patient (*n* = 1834, 15.9%) or the medication error occurred and reached the patient, but it did not cause patient harm (*n* = 1732, 15.0%). The complete categorisation of possible outcomes for patient harm is shown in Table 2. Table 2 also shows possible outcomes for patient harm relating to prescribing, dispensing and administrative electronic systems.

**Table 2:** Severity of medication errors associated with possible outcomes for patient harm (*N* = 11,540)

Patient outcome	n	%
1. Circumstances have the capacity to cause error	117	1.0
2. Error occurred but the error did not reach the patient	1834	15.9
3. Error occurred that reached the patient, but did not cause harm	1732	15.0
4. Error occurred that reached the patient and required monitoring or intervention to confirm no harm	7095	61.5
5. Error occurred that may have resulted in temporary harm and required intervention	673	5.8
6. Error occurred that may have resulted in temporary harm and required prolonged hospitalisation	75	0.6
7. Error occurred that may have resulted in permanent patient harm	8	0.07
8. Error occurred that may have required intervention necessary to sustain life	1	0.009
9. Error occurred that may have contributed to or resulted in the patient’s death	5	0.04
Electronic system	n	%
Prescribing (*n* = 562)		
Patient outcome 1–3	301	53.6
Patient outcome 4–9	261	46.4
Dispensing (*n* = 61)		
Patient outcome 1–3	42	68.9
Patient outcome 4–9	19	31.1
Administering (*n* = 324)		
Patient outcome 1–3	108	33.3
Patient outcome 4–9	216	66.7

### Communication affecting medication errors

Types of communication affecting medication errors were identified for 10,944 (94.8%) medication errors (Table 3). The most common communication types related to health professional misreading or not reading a medication order (*n* = 3726, 32.3%). Poor communication during informal conversations at the bedside (*n* = 2172, 18.8%) and the use of incorrect prescriptions for medication orders (*n* = 1494, 12.9%) were also relatively common.

**Table 3:** Types of communication affecting medication errors (*N* = 11,540)

Type of communication	n	%
Misread or unread order	3726	32.3
Informal bedside communication (poor/lack of communication at bedside)	2172	18.8
Incorrect/inappropriate prescription	1494	12.9
No valid prescription for medication	859	7.4
Incomplete order	576	5.0
Unsigned order or signed in the wrong place therefore appearing as though unsigned	371	3.2
Misread or unread label/container	329	2.9
Multiple or duplicate medication charts in use, such as one paper chart and one electronic chart or two of same kind of chart	274	2.4
Poor or lack of communication within handover	222	1.9
Poor communication within medical record documentation	207	1.8
Duplicate order on medication chart	195	1.7
Ambiguous order	150	1.3
Misinterpreted order	141	1.2
Prescribed in the wrong place of chart or on the wrong type of chart	73	0.6
Poor telephone communication	67	0.6
Illegible handwriting	44	0.4
Wrong unit of measurement placed on medication order	25	0.2
Poor/lack of communication during ward round	14	0.1
Wrong placement of decimal point on medication order	5	0.04
No direct communication cause identified	596	5.2
**Medication errors due to electronic systems**		
Prescribing processes using electronic systems	562	4.9
Dispensing processes using electronic systems	61	0.5
Administration processes using electronic systems	324	2.8

### Human factors and contributing factors of medication errors

Human factors affected the occurrence of medication errors (Table 4). For many medication errors, common human factors related to performance deficit of the health professionals involved with the medication errors (8462, 73.3%) and inadequate screening and assessment of patients (*n* = 1701, 14.7%). While inadequate screening and assessment may be perceived as a type of performance deficit, when it was identified, it was categorised within the specific grouping of inadequate screening and assessment to determine how frequently this factor occurred [20]. Medication errors were also affected by health professional stress (*n* = 62, 0.5%), intimidating behaviour (*n* = 58, 0.5%) and fatigue (*n* = 13, 0.1%). Contributing factors for medication errors related to health professionals not following policies and procedures correctly (*n* = 9510, 82.4%), problems relating to patients’ movements across transitions of care (*n* = 494, 4.3%) and lack of adequate floor stock of various medications (*n* = 424, 3.7%). When patients moved across transitions of care, high-alert medications were commonly associated with medication errors including intravenous antimicrobial and opioid medications, insulin infusions, parenteral potassium infusions, and anticoagulants. Of patients moving across transitions of care (*n =* 494), there were 151 medication errors (30.6%) that had a severity of patient harm score of 1–3, indicating that no harm occurred, while 343 medication errors (69.4%) had a severity of patient harm score of 4–9, indicating that possible or probable harm occurred.

**Table 4:** Human factors and contributing factors identified from medication errors (*N* = 11,540)

Type of human factor	n	%
Performance deficit	8462	73.3
Inadequate screening/assessment of patient	1701	14.7
Knowledge deficit	548	4.7
Error in dispensing/stocking	417	3.6
Miscalculation of dose or infusion rate	154	1.3
Failure to activate delivery system properly	68	0.6
Stress	62	0.5
Intimidating behaviour	58	0.5
Wrong amount of active medication used	39	0.3
Fatigue or lack of sleep	13	0.1
Wrong diluent used for infusion	9	0.08
Wrong amount of diluent used	6	0.05
Wrong medication added to infusion	3	0.03
**Type of contributing factor**		
Policies and procedures	9510	82.4
Patient transfers	494	4.3
Lack of floor stock	424	3.7
Insufficient or incorrect counselling to patients and family	336	2.9
Covering personnel	299	2.6
Frequent interruptions and distractions	214	1.9
High patient acuity and workload	186	1.6
Lack of staff	42	0.4
Inadequate training	35	0.3

### Patient and family involvement

Patients or families were involved in the detection of 1100 (9.5%) medication errors. The types of medications with which patients and families alerted health professionals about actual or potential medication errors comprised patients’ regular medications, such as those for cardiovascular, respiratory and musculoskeletal conditions. However, patient and family alerts also occurred for those medications started during patients’ hospitalisation, including antimicrobials, blood and electrolyte products, anaesthetics and analgesics following surgery. Patient and family involvement was identified in 5% (28/562) of medication errors relating to prescribing electronic systems, in 4.9% (3/61) of medication errors arising from dispensing electronic systems, and in 6.8% (22/324) of medication errors occurring in administering electronic systems. With increasing age, there were reduced odds that patients were involved in identifying the medication error (OR = 0.991, 95% CI 0.988–0.993, *p* < 0.001). Informal bedside conversations demonstrated extremely high odds that patients and families were involved in identifying a medication error (OR = 6.012, 95% CI 5.280–6.844, *p* < 0.001). Female patients compared to male patients showed increased odds of being involved in the detection of medication errors (OR = 1.389, 95% CI 1.221–1.579, *p <* 0.001).

### Associations of person-related, environment-related and communication-related factors for medication errors with severity of harm

Results for univariate logistic regression of diverse person-related, environment-related and communication-related factors are shown in Table 5. Univariate logistic regression identified nine explanatory factors that significantly predicted associations with possible or probable harm compared with no harm from medication factors. With regards to person-related factors, when the individual responsible for the medication error was a doctor, pharmacist, or patient or family member, there were reduced odds of medication errors associated with possible or probable harm, compared with when a nurse or midwife was responsible. Insufficient medication counselling of patients was associated with increased odds of possible or probable harmful medication errors. In regards to environment-related factors, problems with interruptions and the use of covering health professionals increased odds of possible or probable harm. With respect to communication-related factors, misread or unread orders, and poor or no communication during clinical handovers significantly increased odds of possibly or probably harmful medication errors. Conversely, use of prescribing processes with electronic systems, and use of dispensing processes with electronic systems were significantly associated with reduced odds of possibly or probably harmful medication errors, compared to the use of paper-based systems.

**Table 5:** Univariate logistic regression for explanatory factors of medication errors associated with causing possible or probable harm (*N* = 11,540)

	Reference level	Comparator level	Odds ratio	95% confidence intervals	*p*-value
**Person-related factors**					
Individual responsible for the medication error	Nurse or midwife	Doctor	0.446	0.407–0.489	< 0.001
	Nurse or midwife	Pharmacist	0.286	0.236–0.347	< 0.001
	Nurse or midwife	Patient or family	0.761	0.586–0.987	0.039
Involvement of patient or family in identifying medication error	No	Yes	1.024	0.896–1.170	0.730
Insufficient counselling of patient	No	Yes	2.619	1.943–3.529	< 0.001
Age of patient	Younger than 65 years	65 years and above	1.504	1.390–1.627	< 0.001
**Environment-related factors**					
Patient movement across transitions of care	No	Yes	1.068	0.878–1.298	0.511
Interruptions	No	Yes	1.940	1.382–2.725	< 0.001
Covering personnel	No	Yes	1.855	1.396–2.464	< 0.001
Medication checking	Single checked	Double checked	1.083	0.993–1.180	0.071
**Communication-related factors**					
Misread or unread order	No	Yes	2.562	2.333–2.812	< 0.001
Informal bedside conversations	No	Yes	1.003	0.908–1.109	0.951
Error related to clinical handover	No	Yes	1.433	1.054–1.948	0.022
Error related to prescribing processes using electronic systems	No	Yes	0.386	0.325–0.458	< 0.001
Error related to electronic dispensing processes	No	Yes	0.210	0.122–0.362	< 0.001
Error related to use of electronic systems to administer the medication	No	Yes	0.936	0.740–1.183	0.579

Table 6 shows the results of multivariable logistic regression for person-related, environment-related and communication-related factors in regards to the occurrence of medication errors producing possible or probable harm. Eleven explanatory variables demonstrated significant associations with possible or probable harmful medication errors. For person-related factors, doctor, pharmacist, or patient or family responsibility for medication errors compared to nurse or midwife responsibility showed reduced odds of possible or probable harmful medication errors. Insufficient counselling of patients was associated with increased odds of possible or probable harm. With respect to environment-related factors, the presence of patient movements across transitions of care, interruptions during any stages of medication management and covering personnel all increased the odds, while double-checking of medications compared to single-checking was associated with reduced odds of possibly or probably harmful medication errors. In relation to communication-related factors, misread or unread medication orders, informal bedside conversations, and problems with clinical handovers all increased the odds, while the presence of electronic prescribing systems and electronic dispensing systems reduced the odds of possibly or probably harmful medication errors.

**Table 6:** Multivariable logistic regression model for explanatory factors of medication errors associated with causing possible or probable harm (*N* = 11,540)

	Reference level	Comparator level	Odds ratio	95% confidence intervals	*p*-value
**Person-related factors**					
Individual responsible for the medication error	Nurse or midwife	Doctor	0.690	0.618–0.771	< 0.001
	Nurse or midwife	Pharmacist	0.327	0.267–0.401	< 0.001
	Nurse or midwife	Patient or family	0.641	0.472–0.870	0.001
Involvement of patient or family in identifying medication error	No	Yes	0.924	0.789–1.083	0.331
Insufficient counselling of patient	No	Yes	3.511	2.512–4.908	< 0.001
**Environment-related factors**					
Patient movement across transitions of care	No	Yes	1.461	1.190–1.793	0.001
Interruptions	No	Yes	1.432	1.012–2.027	0.043
Covering personnel	No	Yes	1.490	1.113–1.995	0.007
Double and single medication checking	Single checked	Double checked	0.905	0.826–0.991	0.032
**Communication-related factors**					
Misread or unread order	No	Yes	2.411	2.162–2.690	< 0.001
Informal bedside conversations	No	Yes	1.221	1.085–1.373	0.001
Error related to clinical handover	No	Yes	1.559	1.136–2.139	0.006
Error related to prescribing processes using electronic systems	No	Yes	0.580	0.478–0.705	< 0.001
Error related to electronic dispensing processes	No	Yes	0.350	0.199–0.618	0.001
Error related to use of electronic systems to administer the medication	No	Yes	1.214	0.933–1.581	0.149

## Discussion

The interplay of person-related, environment-related and communication-related factors was associated with possible or probable harm caused by medication errors. Multivariable regression showed that medication errors caused by doctors, pharmacists and patients or families compared to those caused by nurses or midwives, the presence of double-checking of medications compared to single-checking, and the presence of electronic systems for prescribing and dispensing were significantly associated with reduced odds of possibly or probably harmful medication errors. Conversely, multivariable regression demonstrated that insufficient counselling of patients, patient movement across transitions of care, interruptions, the presence of covering personnel, misread or unread orders, informal bedside conversations, and problems with clinical handovers were associated with increased odds of medication errors causing possible or probable harm.

Patients and families were involved in detecting about 1 in 10 medication errors. This result indicates the important role that patients and families have in understanding patients’ medication regimen. This detection comprised a variety of medication errors reported, which provides some indication of the wide breadth of patient and family understanding about medications used in the clinical settings. Past research has shown health professionals perceive that patients and families do not need to know about medication changes during hospitalisation, because it is likely that these medications may not be continued following discharge [21, 22]. However, the current study shows that patient and family understandings of regular and newly-commenced medications can help to alert health professionals of any potential problems associated with their use. This audit identified that patient and family involvement tended to be much higher during informal bedside conversations compared to the use of other communication channels. In particular, seeing patients at the bedside may have helped nurses to confirm medication details during their administration of medications with patients and their families [23]. Conversely, while patients and families played a role in informing health professionals about medication errors during informal bedside conversations, this communication channel was associated with medication errors with possible and probable harm. Informal bedside conversations were also where interruptions between health professionals occurred. These activities could have contributed to possible or probable harm relating to medication errors. It is also possible that patients and families detected the error but the error had already occurred, and that they did not necessarily prevent the error.

Patient movement across transitions of care was associated with possible or probable harm with medication errors. These transitions included patient transfers to and from hospital, as well as those happening internally between clinical settings. Of concern, was the use of high-alert medications, which may have contributed to patient harm occurring at transitions of care. Individuals responsible for causing medication errors across transitions of care have largely comprised doctors and nurses, and to a lesser extent pharmacists. However, while policy guidelines indicate the importance of all professional groups collaborating to prevent and resolve medication errors at transitions of care [24], research has demonstrated that nurses, doctors and pharmacists themselves perceive that this is pharmacists’ responsibility [12, 25]. Rather, interdisciplinary initiatives between various health professional disciplines across transitions of care can help to reduce medication errors [26–29].

For the study, the presence of double-checking and single-checking was identified by specific notation found in each medication error report. For the purpose of this study, there was no attempt to further break down the various meanings of double-checking. A review of recent literature shows that double-checking is a complex process with many possible meanings. A vital element of double-checking is the independent nature of this process, whereby both the requesting nurse and the checking nurse perform a check individually without sharing information with each other. Conversely, a less desirable approach is primed double-checking where the requesting and checking nurses share information with each other. Double-checking can be a mandated process, where it is regarded as an authoritative command while when double-checking is a non-mandated process, it is considered a voluntary activity [30]. Further research should consider these various meanings of double-checking to determine how they relate to possible or probable harm with medication errors.

Interruptions were shown to be associated with medication errors causing possible or probable harm. Past research has shown that interruptions may also be related to non-patient activities, which increases the complexity of identifying possible strategies for reducing their occurrence [31]. Many strategies have been trialled to address interruptions including interruption-free zones, posters, wearing of vests and behavioural interventions with mixed success [31–33]. Despite past work indicating nurses’ awareness of how interruptions can affect patient safety, nurses have highlighted barriers to change in the ways they react to interruptions [34]. Interviews with nurses have indicated they feel pressure to complete clinical tasks within specified time periods, and in the presence of limited staffing, nurses perceived they could be distracted from their medication activities. In the current study, interruptions were associated with unread or misread medication orders, which subsequently led to increased medication errors relating to patient harm. Another complexity relates to nurses’ perceptions of whether interruptions are predictable or unpredictable. Unpredictable interruptions have been shown to affect nurses’ views about whether interruptions need to be addressed immediately. Predictable and unpredictable causes for interruptions compete for nurses’ time, making it difficult for nurses to consistently meet patient needs [32]. By using redesigned patient care models, it may be possible for nurses to segregate their work based on predictability about clinical tasks, thereby enabling them to complete activities in a timely manner [35].

Informal bedside conversations and poor or lack of communication during clinical handover were associated with increased risk of medication harm. Possible reasons for lack of effective communication about medications in these communication forums related to health professional concerns about relaying confident or sensitive information at the bedside [23]. Presence of electronic prescribing and dispensing systems enabled reduction of harmful medication errors. This reduced harm may have been due to enhanced legibility of medication orders. Nevertheless, these electronic systems were still associated with medication errors as a result of human errors relating to incorrect selection of medications from drop-down menus, or by overriding alarms in the presence of contraindicated medications or incorrect dosing [36]. A key ongoing challenge with electronic systems is their need to be integrated, to enable efficient exchange of patient data between primary care practitioners, hospital care, specialist care and shared personal health records [37]. Enabling patients and families to access their own records in electronic systems is another challenge, but it has a positive effect on medication safety [38].

### Limitations

The study involved a retrospective analysis of reported medication errors. It is possible that various complexities of clinical practice may not have been captured in the information provided by health professionals to the online reporting form. No information was available about years of experience and positions of health professionals responsible for the medication errors or about the demographic characteristics of patients, aside from their age and gender. Patient characteristics such as those whose preferred language spoken is not English, those who are socially disadvantaged or who have low health literacy, or those who have care provided by multiple medical specialties, may have provided additional insights on particular aspects affecting patient involvement in detection of medication errors. Nevertheless, rich descriptions of medication errors were captured from the reporting systems of public and private hospitals, which enabled determination of how person-related, environment-related and communication-related factors were associated with the severity of medication errors. Analysis was completed on all medication errors reported over an 18-month period, which comprised over 11,000 medication errors. The confidence intervals for the various explanatory factors examined were relatively small, leading to precise estimates of their true effects. Australian hospitals use a form of voluntary reporting of medication errors, which means that health professionals were more likely to provide in-depth information about medication errors as the focus was on helping other health professionals to avoid similar errors. These voluntary programs may have encouraged health professionals to report near misses and errors that were unlikely to cause harm. Such reporting may be associated with diluting of reported information, such that critical information could be lost. Furthermore, health professionals may not have reported medication errors in the voluntary system. Some health disciplines such as nurses may have been more likely to report errors of omission or near misses than doctors. In this study, aside from near misses and medication errors that were unlikely to cause harm, health professionals reported medication errors associated with temporary or permanent harm. It also needs to be acknowledged that incident reports can only occur when a medication error is observed or identified by health professionals, or patients and their families in real time or at least during the patients’ hospital stay. This reporting often does not happen as many medication errors go unnoticed.

### Implications for practice

Patients and families need to be engaged in discussions about medications. Multiple teachable opportunities present during bedside conversations, admission and discharge consultations and medication administration activities could improve patient and family understanding. Patient counselling needs to be more targeted and structured in efforts to reduce medication errors associated with possible or probable harm. Greater attention could be given in the curricula of health professional pharmacology courses about strategies for enabling targeted and structured patient counselling [39]. Patients and families were rarely involved in the detection of medication errors comprising electronic systems for prescribing, dispensing and administration when compared to paper systems. In view of the steady move of hospital networks from paper-based to electronic systems, more attention needs to be placed on strategies in which patients and families could be encouraged to be involved in decision making with electronic systems. Such strategies could include sharing the computer screen with patients and families during medication discussions at ward rounds and facilitating use of patient portals. Greater efforts are needed to enhance reporting of medication errors by doctors, which could involve facilitating discussions about addressing tensions between ensuring a duty of care in acknowledging the occurrence of a medication error and experiencing a desire to protect themselves from accusations of negligence. There should be protected time allocated to key individuals participating in clinical handover and to ensuring all relevant changes to medications are provided in a clear and structured manner. When patients are transferring between settings, it is important that health professionals in the oncoming team are provided with medication details that are likely to impact patient care following the transfer. Informal bedside conversations should be followed up with documentation of plans of care relating to managing medications within the medical record. Patients and families should be provided with these plans to ensure they are informed about the outcomes of informal conversations.

## Conclusions

This study demonstrated complex associations between a wide diversity of person-related, environment-related and communication-related factors on the occurrence of medication errors leading to possible and probable harm. Further research is needed to examine associations between patients’ characteristics and medication-related harm, such as patients whose preferred language spoken is not English, who are socially disadvantaged, who have low health literacy, and who have care provided by multiple medical specialties. Consideration of the characteristics of health professionals such as their years of experience and familiarity with the work environments, and of how these characteristics affect their communication with colleagues, patients and families, would provide further insights into medication errors leading to possible or probable harm.

## Data Availability

The datasets generated and analysed during the current study are not publicly available due the extremely sensitive nature of these data relating to the occurrence of medication errors and patient outcomes. Ethics approval was granted on condition that these data are not made public. The corresponding author is willing to discuss issues relating to the data with interested individuals on reasonable request.
